# Prevalence of depression and anxiety among elderly primary care patients in Palestine

**DOI:** 10.3389/fpsyt.2023.1291829

**Published:** 2024-01-19

**Authors:** Beesan Nader Maraqa, Zaher Nazzal, Suha Hamshari, Barlant Alutt, Ekram Rishmawi, Abdallah Qawasmeh

**Affiliations:** ^1^College of Medicine, Hebron University, Hebron, Palestine; ^2^Faculty of Medicine and Health Sciences, An-Najah National University, Nablus, Palestine; ^3^Department of Family and Community Medicine, Faculty of Medicine and Health Sciences, An-Najah National University, Nablus, Palestine

**Keywords:** depression, anxiety, elderly, primary health, mental health

## Abstract

**Background and objectives:**

Depression and anxiety are common mental health disorders among the elderly worldwide. In this study, we estimated the prevalence of depression and anxiety and related risk factor among elderly attending Primary Health care (PHC) centers in Palestine.

**Methods:**

A cross-sectional study was conducted from February to July 2021 with a sample size of 380 participants aged ≥60 attending PHC centers in West Bank, using an interviewer-administered questionnaire. We used the Geriatric Depression Scale-15 and the Geriatric anxiety Scale to screen for depression and anxiety, respectively. Logistic regression models were used to identify predictors of depression and anxiety.

**Results:**

The prevalence of depression and anxiety was 41.1 and 39.2%, respectively. Elderly people living in rural areas (aOR = 2.63; 95% CI: 1.72–4.20), uneducated (aOR = 2.92; 95% CI: 1.41–6.13), and without monthly income (aOR = 3.42; 95% CI: 1.52–7.61) were more likely to have depression. On the other hand, anxiety was independently associated with living in rural areas (aOR = 1.93, 95% CI: 1.23–3.04) and having non-communicable diseases (aOR = 2.01; 95% CI: 1.13–3.49).

**Conclusion:**

Depression and anxiety are common in Palestine, a developing country with a lack of elderly related services. This should be emphasized at the national and regional levels where geriatric health care services are scarce. Such information is required by policymakers and external funding agencies in order to develop future agendas.

## Introduction

Nowadays, the average individual can foresee living well into their sixties and beyond. People over 60 years of age are a growing proportion of the general population. In 2019, the global population of adults aged 60 and over topped one billion. The forecast estimates 1.4 billion people in 2030 and 2.1 billion in 2050 (1). Addressing the specific healthcare needs of the elderly population is essential in health aging (2). While older individuals in the developed world have slightly better health than previous generations, good health later in life is not fairly distributed. About 5% of the population in Palestine consists of individuals aged 60 (3).

Mental health is an important part of living well. Care services are still below optimal in low to middle-income countries. According to World Health Organization 14% of adults 60 and older suffer a mental health condition (4). A recent pooled analysis of depression in a systemic review study on prevalence among the elderly revealed an average predicted prevalence of 31.7% (95% confidence interval (CI): 27.9–35.6). In the subgroup analysis, the pooled prevalence was higher in developing countries, reaching almost 40.8%. The associated risk factors could be being older, female, single or divorced, having a low educational level (low or nonexistent), family history of significant life events (i.e., family death) and having chronic diseases (i.e., diabetes and heart diseases) (5). Along with depression, anxiety is a prevalent mental health condition in the elderly. Community samples revealed up to 15% suffered from anxiety, while clinical settings showed up to 28%. It was significantly higher in terms of anxiety symptoms, ranging between 15 and 52.3% in population samples and 15% to 56% in clinical samples (6).

Healthcare practitioners often underdiagnose mental health conditions, and the stigma associated with these conditions inhibits people from getting help. Few studies evaluated common mental health condition among older people in Arab countries (7). The stigma associated with the significant societal implications of mental health condition might be one barrier to diagnosis and treatment (8).

In Palestine, clinicians do not have screening guidelines and rarely use screening instruments. However, numerous visits to primary health care centers (PHC) with physical symptoms are indicators of mental health conditions that frequently go untreated (9). Furthermore, Palestine faces distinct difficulties with movement restrictions imposed by the Occupation and a dearth of mental health services, making this study crucial. Therefore, our study aim is to determine the prevalence of depression and anxiety among elderly adults presenting to PHC in the Occupied West Bank and factors that influence those mental health diagnoses.

## Methods

### Design and study population

We conducted a cross-sectional study using an interview-administered questionnaire. Adults aged 60 years and older who attended the three main PHC centers in the West Bank of Palestine were asked to participate between February to July 2021. The sample size was 380, as calculated by Epi Info 6.0, at an 80% power level and a 95% confidence level (10).

### Measures

An interviewer-administered questionnaire consisted of 3 sections:

(1) Patient information: a. sociodemographic variables which includes: Gender, age (60–64; 65–70; >70), Marital status (married; single: single or widowed), residency (urban; rural), educational level (uneducated+ primary school; middle + high school; university), living conditions (alone; not alone), professional status (employed; retired; not employed) and source of income (no income/social/saving; from family; retirement salary or private job).

b. Medical history of non-communicable diseases (NCD) such as hypertension, diabetes mellitus, cancer, and ischemic heart disease as reported by the particpants. If a patient presents with one or more NCDs, they are classified as having NCDs.

(2) GDS-15: a short version of the Geriatric Depression Scale that is used for the purpose of assessing depression symptoms in older adults. The Geriatric Depression Scale (GDS) is a measurement tool that assigns scores ranging from 0 to 15. A score of 0 on the GDSsuggets the absence of depressive symptoms, scores between 5 and 8 suggest mild depressive symptoms, scores between 9 and 11 indicate moderate depressive symptoms, and scores between 12 and 15 indicate severe depressive symptoms (11–13). Depressive symptoms was suggested by a cut-off value of six or higher (11, 12).

(3) Geriatric anxiety Scale (GAS): the scoring system used in this study categorizes individuals based on their level of anxiety symptoms. A score ranging from 0 to 11 is indicative of minimum anxiety symptoms, a score between 12 and 21 suggests mild anxiety symptoms, Scores of 22 to 27 suggests moderate anxiety symptoms and scores of 28 and above indicates severe anxiety symptoms (14, 15). To categorize anxiety into two groups, a cut-off score of 16 was chosen based on the literature. Those with a score greater than 16 were have no anxiety, while those with a score less than or equal to 16 were classified as have anxiety (16).

Arabic versions of both the used scales have a high level of reliability (12, 17).

#### Stastical analysis

The analysis was conducted using IBM SPSS Statistics for Windows, Version 20.0 (IBM Corp., Armonk, NY: IBM Corp). The demographic and clinical features of the subjects were described using descriptive statistics (means, standard deviations for continuous variables, and frequency distributions and proportions for categorical variables). Additionally, “chi-square test” was employed to determine any significant relationships between the groups. We employed binary logistic regression in a multivariate analysis to account for potential confounding factors. The models incorporated all variables identified as significant in the univariate analysis, as well as those with a *p* value below 0.1.

### Ethical considerations

All methods involving human participants in this study were conducted per ethical research standards. The study was conducted in conformity with the ethical norms of An-Najah National University (ANNU). The Ministry of Health approved authorization for the study to be conducted in PHC settings, and participants were approached and invited voluntarily to participate. Participants were assured of their confidentiality and anonymity.

## Results

Of the 447 questionnaires distributed, 380 patients agreed to the interview, representing an 85% response rate. Just over half (52.1%) were male, most (76.6%) were married, two thirds (69.9) were between 60 and 70 years of age. Sociodemographics are presented in Table 1.

**Table 1:** Sociodemographic characteristics of the studied sample (*n*=380).

Variables	*N* (%)
**Age**	
60–64 years	147 (38.6)
65–70 years	118 (31.1)
>70 years	115 (30.3)
**Gender**	
Male	198 (52.1)
Female	182 (47.9)
**Marital status**	
Married	291 (76.5)
Single	20 (5.3)
Widowed	69 (18.2)
**Residency**	
Urban	172 (45.3)
Rural	208 (54.7)
**Educational level**	
Uneducated +primary school	157 (41.3)
Middle +high school	134 (35.3)
University	89 (23.4)
**House setting**	
Alone	40 (10.5)
Not alone	340 (89.5)
**Professional status**	
Employed	59 (15.6)
Retired	91 (23.9)
Not employed	230 (60.5)
**Source of income**	
No Income or social, or savings	55 (14.5)
From Family	148 (38.9)
Retirement salary or private job	177 (46.6)
Non-communicable disease (Yes)	298 (87.4)
Hypertension	205 (53.9)
Diabetes	161 (57.6)
IHD	102 (26.8)
Cancer	15 (3.9)

IHD, ischemic heart disease.

### Prevalence of depressive and anxiety symptoms

**Figure 1:**
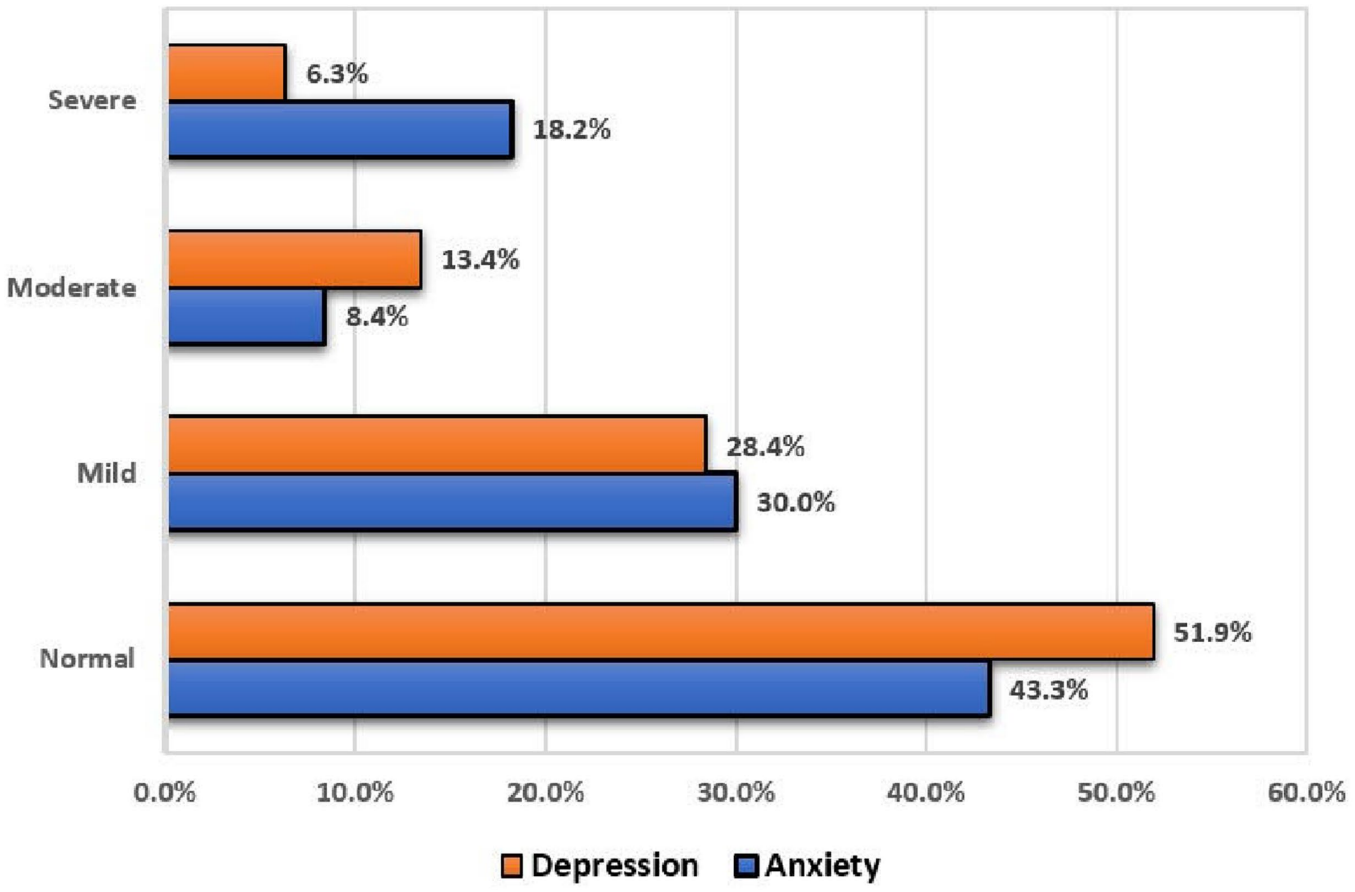
The frequency of depressive symptoms and anxiety symptoms among study participants.

Among the participants, 156 [41.1% (95%CI: 36.1–46.2%)] were found to have depressive symptoms, with mild depressive symptoms accounting for 28.4%, moderate depressive symptoms accounting for 13.4%, and severe depressive symptoms accounting for 6.3%. Anxiety symptoms were reported among 149 participants [39.2% (95%CI: 34.3–44.3%)] with mild anxiety symptoms accounting for 30.0%, moderate anxiety symptoms accounting for 8.4%, and severe anxiety symptoms accounting for 18.2% (Figure 1).

Being older, living in rural areas, lower educational level, unemployed, and no source of income were significantly associated with depressive symptoms (Table 2).

**Table 2:** Association between sociodemographic and clinical factors, and depressive symptoms.

	Depressive symptoms			
Variables	Yes (*n*=156)	No (*n*=224)	*p* value*	OR (95%CI)
**Age**				
60–64 years	64 (43.5%)	83 (56.5%)		1
65–70 years	64 (43.6%)	77 (65.4%)	0.24	0.69 (0.42–1.34)
>70 years	51 (44.3%)	64 (55.7%)		1.03 (0.63–1.69)
**Gender**				
Male	74 (37.3%)	124 (62.7%)	0.13	1
Female	82 (45.1%)	100 (54.9%)		1.37 (0.92–2.07)
**Marital status**				
Married	112 (38.5%)	179 (61.5%)		1
Single	44 (49.4%)	45 (50.6%)	0.07	1.56 (0.97–2.52)
**Residency**				
Urban	46 (26.7%)	126 (73.3%)	<0.001	1
Rural	110 (52.9%)	98 (47.1%)		3.08 (2.00–4.74)
**Educational level**				
Uneducated + primary school	87 (55.4%)	70 (44.6%)		4.29 (2.38–7.73)
Middle +high School	49 (36.6%)	85 (63.4%)	<0.001	2.00 (1.10–3.66)
University	20 (22.5%)	69 (77.5%)		1
**Living conditions**				
Alone	22 (55.0%)	18 (45.0%)	0.06	1.88 (0.97–3.64)
Not alone	134 (39.4%)	206 (60.6%)		1
**Professional status**				
Employed	16 (27.1%)	43 (72.9%)		1
Retired	25 (27.5%)	66 (72.5%)	<.001	1.02 (0.488–2.13)
Not employed	115 (50.0%)	115 (50.0%)		2.69 (1.43–5.04)
**Source of income**				
No income/social/savings	115 (50.0%)	18 (32.7%)		6.6 (3.00–10.94)
From family	72 (48.6%)	76 (51.4%)	<.001	2.6 (1.65–4.16)
Retirement salary or private job	77 (26.6%)	130 (73.4%)		1
**Non-communicable disease** ^†^				
Yes	130 (43.6%)	168 (56.4%)		1.67 (0.992–2.80)
No	26 (31.7%)	56 (68.3%)	0.05	1

*Chi-squared test, †Non-communicable disease, OR: odds ratio, CI: confidence interval.

In contrast, being older, female, single, living in rural areas, and having lower educational level were significantly associated with higher level of anxiety symptoms. Furthermore, older people who live alone, are unemployed, have no income, or have a non-communicable disease were found to have significantly more anxiety symptoms (Table 3).

**Table 3:** Association between sociodemographic and clinical factors, and anxiety symptoms.

	Anxiety symptoms			
Variables	Yes (*n*=149)	No (*n*=231)	*p* value_*_	OR (95%CI)
**Age**				
60–64 years	60 (40.8%)	87 (59.2%)		1
65–70 years	37 (31.4%)	81 (68.6%)	0.08	0.66 (0.40–1.10)
>70 years	52 (45.2%)	63 (54.8%)		1.20 (0.73–1.96)
**Gender**				
Male	62 (31.3%)	136 (68.7%)	0.001	1
Female	87 (47.8%)	95 (52.2%)		2.01 (1.32–3.06)
**Marital status**				
Married	103 (35.4%)	188 (64.4%)	0.01	1
Single	46 (51.3%)	43 (48.3%)		1.95 (1.21–3.16)
**Residency**				
Urban	50 (29.1%)	122 (70.9%)	<0.001	1
Rural	99 (47.6%)	109 (52.4%)		2.22 (1.45–3.40)
**Educational level**				
Uneducated +primary school	81 (51.6%)	76 (48.4%)		2.89 (1.64–5.07)
Middle +high School	44 (32.8%)	90 (67.2%)	<0.001	1.32 (0.73–2.39)
University	24 (27.0%)	65 (73.0%)		1
**Living conditions**				
Alone	22 (55.0%)	18 (45.0%)	0.03	2.05 (1.06–3.97)
Not alone	127 (37.4%)	213 (62.6%)		1
**Professional status**				
Employed	15 (25.4%)	44 (74.6%)		1
Retired	25 (27.5%)	66 (72.5%)	<0.001	1.11 (.53–2.34)
Not employed	109 (47.4%)	121 (52.6%)		2.64 (1.39–5.01)
**Source of income**				
No income /social /savings	32 (58.2%)	23 (41.8%)		3.63 (6.81–3.52)
From Family	68 (45.9%)	80 (54.1%)	<0.001	2.22 (1.40–3.52)
Retirement salary or Private Job	49 (27.7%)	128 (73.2%)		1
**Non-communicable disease**				
Yes	129 (43.3%)	169 (56.7%)	0.001	2.37 (1.36–4.11)
No	20 (24.4%)	62 (75.6%)		1

*Chi-squared test, OR: odds ratio, CI: confidence interval.

Multivariable analysis showed that being older people, living in rural areas (aOR = 2.63; 95% CI: 1.72–4.20), lower education (aOR = 2.92; 95% CI:1.41–6.13) and no income (aOR = 3.4; 95% CI: 1.52–7.61) were more likely to have depressive symptoms. On the other hand, anxiety symptoms among older people were found to be associated with living in rural areas (aOR = 1.93; 95% CI: 1.23–3.04) and among those with a non-communicable disease (aOR = 2.01; 95% CI: 1.13–3.49) (Table 4).

**Table 4:** Multivariate results of the association between sociodemographic and clinical factors, and depressive and anxiety symptoms.

	Depressive symptoms	Anxiety symptoms
Variables	Adjusted OR (95%CI)	Adjusted OR (95%CI)
**Age (years)**		
60–64 years^†^	1	1
65–70 years	1.48 (0.83–2.72)	0.57 (0.33–1.09)
>70 years	0.841 (0.46–1.48)	0.87 (0.45–1.54)
**Sex**		
Male^†^	1	1
Female	0.750 (0.43–1.32)	1.31 (0.77–2.87)
**Marital status**		
Married^†^	1	1
Single	1.12 (0.62–2.04)	1.30 (0.72–2.19)
**Residence** ^†^		
Urban^†^	1	1
Rural	2.63 (1.72–4.20)	1.93 (1.23–3.04)
**Educational level**		
Uneducated or primary school	2.92 (1.41–6.13)	1.62 (0.82–3.60)
Middle or high school	1.51 (0.77–3.09)	0.94 (0.48–1.91)
University^†^	1	1
**Professional status**		
Employed^†^	1	1
Retired	1.54 (0.65–3.43)	1.20 (0.51–2.58)
Not employed	1.61 (0.62–4.03)	1.52 (0.59–3.61)
**Source of income**		
No Income/social/savings	3.42 (1.52–7.61)	2.04 (0.9–4.41)
From Family	1.50 (0.76–2.92)	1.12 (0.56–2.13)
Retirement salary or private job^†^	1	1
**Non-communicable disease**		
Yes	1.37 (0.75–2.50)	2.01 (1.13–3.49)
No^†^	1	1

^†^Reference level, OR: odds ratio, CI: confidence interval.

## Discussion

In our study, we found that 41.1% suffer from depressive symptoms, and 39.2% from anxiety symptoms. These findings corroborate a meta-analysis that assessed the global prevalence of depression to be 31.7% and 40.7% in developing countries (5). In this study, Cronbach alpha for depressive symptoms items is 0.83 and anxiety symptoms items is 0.92, which shows excellent reliability. Research conducted in nearby Egypt with similar culture reported a prevalence of depression and anxiety symptoms of 37.5% and 14.2%, respectively (7).

To our knowledge, this is the first study examining the prevalence of depression and anxiety in adults over 60 years seeking care in primary care centers in Palestine. Consistent with other studies, physical illness is a common risk factor for mental illness, particularly in the elderly (18).

Our results for both anxiety and depressive symptoms were surprising because the female gender and single status, which are considered risk factors (19), are not significant in our sample. In contrast to the findings of an Indian study that demonstrated a positive correlation between aging and the prevalence of depression and anxiety, our analysis did not reveal any significant associations between age and either anxiety or depressive symptoms (20). This is likely due to local culture and traditions that value the elderly and encourage people, particularly women and unmarried women, to assist them financially and socially.

In the relationship between depressive symptoms and education, low education was significantly associated with depressive symptoms in developing and developed countries. In contrast, income was not strongly related to depressive symptoms in low- to middle-income countries, contradicting our findings (21). This could indicate that educated individuals better understand the issue and are more likely to seek medical treatment early. In terms of income, as previously stated, a significant proportion of Palestinian families frequently rely on retired older people financially, increasing pressure and the possibility of economic violence; of course, this association warrants further examination to ascertain the patterns implicit in these complex relationships.

The findings of our investigation indicate a correlation between symptoms of anxiety and the process of aging. This conclusion exhibits a correlation with a prior study that was undertaken in Egypt and China (22, 23). Furthermore, a noteworthy correlation was identified between anxiety symptoms and factors like place of residence, source of income, and educational attainment, which aligns with findings from a previous investigation.

Although there is no substantial association between urban and non-urban residency and symptoms of depression and anxiety,rural–urban differences in mental disorders have piqued the interest of researchers and policymakers involved in mental health care. Due to community ties and social isolation, city inhabitants may be more prone to depressive symptoms than rural residents (24).

Our study limitations include beginning with the global pandemic of COVID-19, which prevented the researchers and patients from accessing the primary healthcare centers. The social desirability bias could be expected in an interview-based questionnaire about sensitive objects because patients tend to answer positively to the questions, but we hypothesize that this may be minimal in our study because elderly people are fully aware of the consequences of their contribution, which may reflect in their quality of care. Furthermore, the tools employed serve the purpose of screening, while additional diagnostic measures should be employed to accurately diagnose depression and anxiety. Nonetheless, this study provides an in-depth assessment of the prevalence of depression and anxiety symptoms among older individuals. Any individuals who screened positive were subsequently referred for further investigation. Finally, this is a cross-sectional study that measures the prevalence in a snapshot of time, and the prevalence may change over time.

## Conclusion

Palestine is classified as a developing nation that faces challenges in providing comprehensive aging-related care, particularly in the area of screening for common ailments prevalent among the senior population, among economically disadvantaged nations like Palestine, the presence of these mental disorders can place a substantial financial and institutional burden on primary healthcare establishments. Depression and anxiety are prevalent conditions that warrant regular screening of elderly individuals in primary healthcare (PHC) institutions. It is recommended that guidelines for screening be implemented and that doctors receive appropriate training to adhere to these guidelines. Also, there is a need to create interventions aimed at the prevention and management of depression. There is a need to establish campaigns aimed at educating the general population about depression. Policymakers and external funding bodies necessitate such information for the purpose of formulating future agendas. It is imperative to prioritize the implementation of referral mechanisms for individuals exhibiting symptoms of moderate to severe depression and anxiety.

## Data availability statement

The original contributions presented in the study are included in the article/supplementary material, further inquiries can be directed to the corresponding author.

## Ethics statement

All methods involving human participants in this study were conducted per ethical research standards. The study was conducted in conformity with the ethical norms of An-Najah National University (ANNU). The Ministry of Health approved authorization for the study to be conducted in PHC settings, and participants were approached and invited voluntarily to participate. Participants were assured of their confidentiality and anonymity. This study was performed in accordance with the ethical standards of the institutional research committee and with the 1964 Helsinki Declaration and its later amendments or comparable ethical standards. It was approved by the Institutional Review Board (IRB) of An-Najah National University.

## Author contributions

BM: Methodology, Software, Writing – original draft, Writing – review & editing. ZN: Conceptualization, Investigation, Methodology, Software, Supervision, Writing – original draft, Writing – review & editing. SH: Conceptualization, Data curation, Formal analysis, Investigation, Methodology, Project administration, Software, Supervision, Validation, Writing – original draft, Writing – review & editing. BA: Writing – original draft, Writing – review & editing. ER: Writing – original draft, Writing – review & editing. AQ: Supervision, Writing – original draft.
